# Fungal Rhino-orbital Cerebritis in a Patient with Steroid-induced Ketoacidosis

**DOI:** 10.5811/cpcem.2018.8.38664

**Published:** 2018-09-18

**Authors:** Carrie Vargo, Beth-Ann Olliviere-Baptiste, Jay M. Brenner, Derek R. Cooney, Elliot Rodriguez

**Affiliations:** SUNY Upstate Medical University, Department of Emergency Medicine, Syracuse, New York

## Abstract

Fungal rhino-orbital cerebritis is a devastating opportunistic invasive disease. Survival requires urgent diagnosis. Thus, all patients at risk who present with rhinosinusitis-type symptoms and have co-morbid conditions that decrease their immunocompetence should trigger the clinician’s consideration of this disease. Treatment includes antifungals and emergent surgical debridement.

## INTRODUCTION

Fungal rhino-orbital cerebritis is an uncommon but devastating opportunistic, invasive fungal infection with a grim prognosis. Survival requires early diagnosis and aggressive treatment with antifungals and surgical debridement. The following case demonstrates the importance of considering this disease as part of the differential diagnosis especially in patients who are immunocompromised. This patient was initially diagnosed with conjunctivitis and then was thought to have developed possible anaphylaxis. Her rapidly progressing physical exam findings helped lead to making the unfortunate diagnosis.

## CASE REPORT

A 52-year-old woman with multiple comorbidities, including obesity and chronic use of prednisone presumably for pulmonary fibrosis, originally presented to an urgent care center two days prior to presenting to our academic hospital and was prescribed polymyxin for presumed conjunctivitis. The patient then presented to our community campus emergency department (ED) because she felt that her “head is swollen and feels like her throat [is] starting to swell.” She believed she was having an allergic reaction; after using her EpiPen® without resolution, she came to the ED to be treated. On initial exam her vital signs were blood pressure 160/90 millimeters of mercury, pulse 120 beats per minute, temperature of 36.8°C, respiratory rate of 22, and oxygen saturation of 100% on room air. She was noted to have posterior oropharyngeal edema; she was treated for possible anaphylaxis but did not improve. During her work-up it was discovered that she was developing acute vision loss in the right eye. She was transferred to our downtown campus ED for ophthalmological specialty evaluation.

Over the course of a few hours, exam findings progressed to include severe bilateral periorbital swelling and severe chemosis. Repeated extraocular motor tests revealed an initial unilateral ophthalmoplegia that later progressed to bilateral cranial nerves III, IV, and VI palsies. Pupillary light-response exam revealed relative afferent pupillary defects suggesting retinal and/or optic nerve involvement. Her labs were consistent with steroid-induced diabetic ketoacidosis (DKA), white blood cell count 18,400 cells per microliter with 96% neutrophils, and acute kidney injury. Computed tomography (CT) only revealed right proptosis, right periorbital soft tissue swelling, and mucosal thickening within all the paranasal sinuses (Image); CT was unable to characterize the cavernous sinus without venous contrast phase.

Ophthalmology was consulted and reported a funduscopic exam that revealed retinal pattern consistent with right eye central retinal artery occlusion. Otolaryngology was consulted and performed a nasal endoscopy in the ED, finding soft black crusting on the septum and turbinates bilaterally with positive potassium hydroxide (KOH) preparation. In consideration of the patient’s clinical presentation, this was presumed to be most consistent with necrosis from invasive mucormycosis. The patient was admitted to the medical intensive care unit and was treated with broad-spectrum antimicrobials, including amphotericin B liposome. Surgical debridement was discussed with the patient and family who were informed of a likely chance of mortality regardless of intervention, considering the likely cavernous sinus involvement. The patient declined surgical intervention, choosing comfort care, and died eight days after admission.

## DISCUSSION

The constellation of cranial neuropathies, periorbital inflammation, necrotic nasal mucosa, positive KOH preparation, steroid-induced immunosuppression, and DKA eventually led to the clinical diagnosis of fungal rhino-orbital cerebritis, most consistent with the classic presentation of rhinocerebral mucormycosis.^1^ Most estimates of incidence of this disease are expected to be underestimated because of the difficulty with diagnosis; the majority of estimates are based on case series.^2^, ^3^ For example, a review of four cases found that it took on average seven days from time of presentation to diagnosis.^4^ No large epidemiologic study in the United States specific to rhino-orbital cerebritis was found on our literature review, but one study in France found only 530 mucormycosis cases between 2001 and 2010, less than half of them involving the rhino-orbito cerebral system.^5^

**CPC-EM Capsule**

What do we already know about this clinical entity?

Fungal rhino-orbital cerebritis is a rare and deadly infection.

What makes this presentation of disease reportable?

The initial misdiagnosis and rapid progression of findings within the course of this patient’s emergency department visit illustrates the difficulty of recognizing this rare infection.

What is the major learning point?

Identifying at-risk patients and performing a thorough ear, nose, throat, and cranial nerve exam may help facilitate an early and accurate diagnosis.

How might this improve emergency medicine practice?

Fungal rhino-orbital cerebritis should be in the differential diagnosis when immunosuppressed patients present with sinusitis-type symptoms.

Our patient’s symptoms initially began with periorbital swelling and conjunctival infection, which was treated as an allergy initially. Indeed, the initial symptoms of rhinocerebral mucormycosis are usually mistaken for bacterial or viral sinusitis or orbital cellulitis. The absence of fevers in up to 50% of cases further contributes to the delay of the correct diagnosis.^1^ Additional considerations in the differential diagnosis would be based on the timing of presentation. Early on, the patient may have little in the way of neurologic findings; thus, infections such as sinusitis, and facial or orbital cellulitis, as well as upper respiratory and maxillary odontogenic infections, would be included in the differential. Non-infectious considerations would include allergic reactions.

If neurologic findings are present, then one must consider intracranial infections, mass lesions and cavernous sinus thrombosis. The diagnosis of invasive fungal rhino-orbito cerebral mucormycosis became only suspected with development of proptosis, chemosis, vision loss and bilateral ophthalmoplegia. Further supportive of the diagnosis was the recognition of her steroid-induced hyperglycemia and acidosis, an environment usually required to allow this ubiquitous fungus to spread from the sinuses to the orbit, invade the orbital musculature, optic nerve or arterial supply, and to cause ophthalmoplegia, central retinal artery occlusion, and afferent pupillary defects, respectively.^1^,^6^,^7^ If bilateral ophthalmoplegia (“bilateral frozen globes”) later develops, or if cranial nerves are involved, as was seen with our patient, it is an ominous sign that the fungus has invaded the cavernous sinus.^1^,^6^

Definitive diagnosis of mucormycosis must be through surgical exploration and biopsy. Typical laboratory studies such as complete blood count, basic metabolic panel, lactic acid and blood gas analysis may help to suggest sepsis or identify co-morbid conditions such as DKA that increase the risk for this disease but would not be diagnostic. Cultures rarely are useful because Mucorales species are ubiquitous in nature and a common contaminate causing frequent false positives; false negatives are also common because the hyphae are fragile and easily crushed during the culturing process. There are no serological studies that can help with rapid diagnosis, which is why up to half of cases aren’t diagnosed until autopsy.^1^

Unfortunately, radiographic studies are often negative or only depict subtle findings such as thickening of the sinus mucosa as was seen in this case (Image).^6^,^8^ When considering this diagnosis, obtaining a CT venography study of the brain may allow for the recognition of cavernous sinus involvement, although in the absence of fever or other infectious clues it would not be definitive proof of septic etiology. Magnetic resonance imaging is an alternative to CT but would take longer to obtain.

To improve chances of successful treatment, the diagnosis must be made as soon as possible, the underlying risk factors such as hyperglycemia or acidemia reversed, and antifungal therapy and surgical debridement must begin immediately.^8^ There are no formal surgical guidelines; however, in many of the reported cases complete orbital exenteration was required, a course that our patient did not pursue.^6^ The antifungal agent of historical choice, presuming mucormycosis, is amphotericin B.^9^–^11^ Case reports suggest that adjunctive hyperbaric oxygen therapy may improve outcomes, possibly by increasing neutrophilic activity.^1^,^12^ In one large epidemiologic review of 196 patients, rhinocerebral mucormycosis had a 62% mortality. Those who received no treatment had only a 3% survival rate.^13^

One limitation to this case report was our inability to obtain additional information about the patient’s initial presentation to an urgent care center two days prior to her presenting to the ED. Based on the history provided to us by the patient and her family, as well as a review of her medications, we were able to determine her diagnosis and treatment prior to our encounter; but it would have been informative to have an objective medical record that included her initial physical exam findings.

## CONCLUSION

In summary, we present a case of fungal rhino-orbital cerebritis, presumably from mucormycosis. Patients with risk factors (e.g., immunosuppression or DKA) who present with symptoms consistent with sinusitis should have a complete head, ears, eyes, nose and throat evaluation, and a thorough cranial nerve exam.^14^ To maximize chance of survival, urgent diagnosis, reversal of underlying predisposing risk factors, prompt antifungal chemotherapy, and surgical debridement are all critical.^4^,^14^

Documented patient informed consent and/or Institutional Review Board approval has been obtained and filed for publication of this case report.

## Figures and Tables

**Image:**
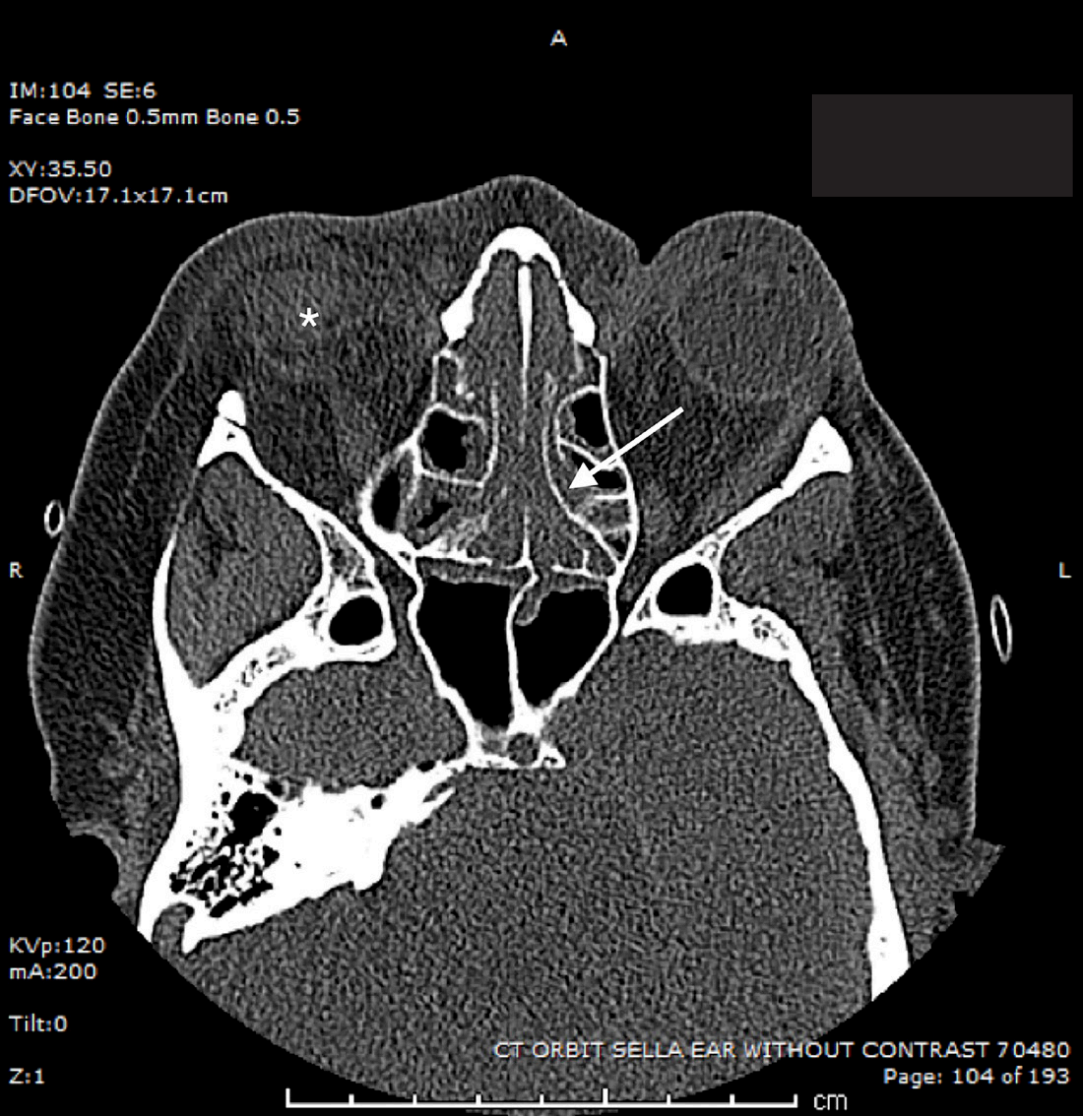
Computed tomography of the orbits, sella and ears without contrast. Right periorbital soft tissue swelling (*), and mucosal thickening within all the paranasal sinuses (arrow).
